# Growth Inhibition of marine microalgae by water-soluble extracts from automobile tires and identifying key chemical contributors to toxicity

**DOI:** 10.1007/s10646-026-03051-6

**Published:** 2026-02-18

**Authors:** Taisei Fujimura, Soichiro Hirashima, Toshimitsu Onduka, Shizuka Ohara, Kazuhiko Takeda, Kazuhiko Koike

**Affiliations:** 1 Graduate School of Integrated Science for Life, Hiroshima University, Higashihiroshima, Hiroshima Japan; 2 Hatsukaichi Branch, Fisheries Technology Institute, Fisheries Research and Education Agency, Hatsukaichi, Hiroshima Japan

**Keywords:** Tire leachate, 1,3-diphenylguanidine (DPG), Diatom, Dinoflagellate, Raphidophyte

## Abstract

Automobile tires are a major source of pollution, releasing complex mixtures of chemicals into aquatic environments. Although the toxicity of tire leachate has been frequently documented, the specific chemical drivers affecting marine microalgae remain largely unidentified. This study investigated the effects of water-soluble leachates from two passenger car tires (Tire A and Tire B) on four marine phytoplankton species: the diatoms *Skeletonema costatum* and *Chaetoceros lorenzianus*, and the flagellates *Chattonella antiqua* and *Karenia mikimotoi*. Leachates from both tires inhibited growth and photosynthesis in all species, with flagellates showing greater sensitivity than diatoms. Tire B leachate exhibited significantly higher toxicity. Chemical analysis using liquid chromatography-mass spectrometry (LC-MS/MS) identified the vulcanization accelerator 1,3-diphenylguanidine (DPG) at 11.37 mg L^− 1^ in the more toxic Tire B leachate, which was not detected in the Tire A leachate. Subsequent exposure experiments with pure DPG confirmed its high toxicity, with 96-hour EC_50_ values for growth inhibition ranging from 0.93 to 2.59 mg L^− 1^. The flagellate *Chat. antiqua* was the most sensitive species. DPG was estimated to contribute 49.4–78.5% of the overall toxicity of Tire B leachate, confirming it as a primary toxicant for the tested species. A potential toxic mechanism is proposed in which the cationic form of DPG electrostatically interacts with negatively charged microalgae cell surfaces, disrupting membrane integrity. These findings identify DPG as a key toxicant from tire wear particles for foundational marine producers, warranting further investigation into its ecological risk.

**Supplementary Information:**

The online version contains supplementary material available at 10.1007/s10646-026-03051-6.

## Introduction

The global expansion of road transportation has led to the massive production and consumption of automobile tires, making them significant sources of environmental pollution. Abrasion at the tire-road interface generates large quantities of Tire and Road Wear Particles (TRWP), a heterogeneous mixture of Tire Wear Particles (TWP) and Roadway Particles (RP) in roughly equal proportions (Panko et al. 2013; Unice et al. 2013). TWP alone can account for up to 30% of total vehicular non-exhaust emissions (Simons 2016). Regional emissions estimates, which typically focus on TWP calculated from tire weight loss, usage, and mileage (Wagner et al. 2018), indicate a significant global environmental burden. Global annual TWP emissions are estimated at 6 million metric tons, with major contributions from the European Union (1.33 million tons) and the United States (1.12 million tons) (Kole et al. 2017; Wagner et al. 2018; Reynolds et al. 2024).

Stormwater runoff transports these particles from roadways into rivers and eventually to coastal marine environments. For instance, roadside stormwater has been found to contain a high abundance of black fragments presumed to be TRWP, comprising up to 64% of total particles (Werbowski et al. 2021). In marine ecosystems, TWPs are a major component of microplastic pollution, accounting for 17.1% of microplastics (< 5 mm) in the Charleston Harbor estuary, USA (Leads and Weinstein 2019), and some estimates suggest they contribute up to 28% of all microplastics in the ocean (Boucher and Friot 2017).

Beyond physical pollution, the chemical constituents leached from tires pose significant toxicological risks (Zhao et al. 2024). Tires contain various organic additives (e.g., antioxidants, vulcanization accelerators) and heavy metals (e.g., zinc) to enhance their durability and performance. These substances can leach into aquatic environments from tire particles (Capolupo et al. 2020; Yang et al. 2022; Jiang et al. 2023; Xu et al. 2024). Furthermore, leached compounds may undergo aquatic transformation, forming byproducts that are sometimes more toxic than their precursors (Johannessen and Parnis 2021; Xu et al. 2024). A striking example is 6PPD-quinone, a transformation product of the antioxidant 6PPD, which has been identified as the lethal agent responsible for mass mortality events in coho salmon (*Oncorhynchus kisutch*) in the U.S. Pacific Northwest (Tian et al. 2021).

Consequently, the ecological risks of tire-derived chemicals to aquatic ecosystems are of growing concern. Adverse effects have been reported across various trophic levels, including copepods, shellfish and macroalgae (Turner and Rice 2010; Capolupo et al. 2020; Bournaka et al. 2023). The toxicity of tire leachates to microalgae, the foundation of aquatic food chains, is also well-documented (Capolupo et al. 2020; Page et al. 2022; Magni et al. 2022; Jiang et al. 2023; Li et al. 2024; Lv et al. 2024). However, the specific chemical drivers of this toxicity remain largely unidentified. While a few causative agents have been implicated for specific organisms—such as 6PPD-quinone for salmon, zinc for a copepod, and cyclic amines for a green microalga (Tian et al. 2021; Yang et al. 2022; Jiang et al. 2023)—knowledge regarding the key toxicants affecting marine microalgae is particularly limited (Capolupo et al. 2020; Page et al. 2022). Disruption at this foundational trophic level could trigger cascading effects throughout the marine ecosystem, threatening vital fishery resources.

To address this knowledge gap, the present study investigated the effects of tire leachates on the growth and photosynthetic activity of four marine microalgae species from three distinct phyla. We further aimed to identify the specific toxicant(s) responsible for the observed impacts. The selected organisms are common inhabitants of the Seto Inland Sea, Japan. Although TRWP pollution has not yet been documented in this specific area, the Seto Inland Sea serves as an ideal model for a semi-enclosed sea under significant anthropogenic pressure from its dense population (35 million people) and extensive industrial activity.

## Materials and methods

### Preparation of tire particle (TP) leachate and culture medium

Two types of new summer passenger car tires were used in this study: Tire A (Sumitomo Rubber Industries, Tokyo, Japan) and Tire B (Toyo Tire Corporation, Hyogo, Japan). Specific product names are not disclosed and are referred to as Tire A and Tire B for clarity. Tire particles (TP) were generated by abrading the tire surface with a coarse metal file. To prepare the leachate, 12 g of TP was suspended in 1.2 L of artificial seawater (Marine Art SF-1, Tomita Pharmaceutical, Tokushima, Japan) in an Erlenmeyer flask, resulting in a concentration of 10 g L^− 1^. The flask was sealed with a silicone stopper and stirred continuously at 600 rpm in a dark chamber maintained at 25 °C for two weeks, following the method of Halsband et al. (2020). After incubation, the mixture was filtered through a glass fiber filter (GF/F; Cytiva, Marlborough, MA, USA) to remove coarse particles, followed by sterile filtration using a 0.2 μm asymmetric polyether sulfone (aPES) filter unit (Cat. No. 597–4520, Thermo Fisher Scientific, Waltham, MA, USA). This final sterile filtrate was designated as the “stock solution”. The culture medium was prepared by enriching this stock solution with nutrients according to the f/2 medium formulation (Guillard 1975), supplemented with sodium selenite (Na_2_SeO_3_; final concentration: 1.0 × 10^− 8^ M) and Tris buffer (final concentration: 2 mM). Sodium selenite was added as an essential micronutrient for marine phytoplankton (Harrison et al. 1988), particularly for the test species *K. mikimotoi* (Hatano and Imai 2010). Tris buffer was used to stabilize the pH throughout the experiment, eliminating potential artifacts from pH fluctuations during the leaching and exposure process. An aliquot of the stock solution, collected prior to nutrient addition, was stored at -20 °C for chemical analyses.

### Effects of tire leachate on marine microalgae growth

Four marine microalgae species were selected for this study: the diatoms *Skeletonema costatum* and *Chaetoceros lorenzianus*, the raphidophyte *Chattonella antiqua*, and the dinoflagellate *Karenia mikimotoi*. All strains were originally isolated from the Seto Inland Sea, a region where they are dominant species. Cultivation, including pre-culturing, was conducted in a temperature-controlled incubator maintained at 25 ± 1 °C under a 12:12 h light: dark cycle with a light intensity of 120–150 µmol photons m^− 2^ s^− 1^. The culture temperature was selected because it represents the optimal growth condition for the test species ensuring that the observed effects were primarily attributable to the tire leachate rather than thermal stress. Stock cultures were maintained in modified f/2 medium prepared with natural seawater collected from offshore of the Sea of Japan to minimize potential low levels of anthropogenic chemical contamination.

The effects of the two tire leachates (Tire A and Tire B, prepared as previously described) on the growth rates of the four microalgae species were evaluated based on the International Organization for Standardization (ISO) 10253 (ISO 2024). However, the validity criterion of the guideline, that the control cell density must increase by a factor of at least 16 during the test period, was not met. This is because the test algae used in this study were originally isolated from our study sites and exhibited slow growth rates that could not intrinsically meet the criterion.

Experiments were conducted in 24-well plates (EZVIEW™ Glass Bottom Culture Plates LB, Iwaki, Tokyo, Japan) in a total volume of 1 mL per well. A dilution series of above-mentioned tire leachate stock solution (equivalent to 10 g L^− 1^ TP, prepared in artificial seawater) was prepared. The stock solution was first diluted 1:1 with modified f/2 medium (based on artificial seawater) to obtain a concentration equivalent to 5 g L^− 1^ TP. This working solution was then serially diluted (1:1 dilutions) to yield concentrations equivalent to 2.5 and 1.25 g L^− 1^ TP.

For the exposure experiments, 200 µL of exponentially growing microalgae culture was added to each designated well. Subsequently, 800 µL of each working solutions (corresponding to 10, 5, 2.5, and 1.25 g L^− 1^ TP, as mentioned above) was added to triplicate wells for each concentration, resulting in final exposure concentrations of 8, 4, 2, and 1 g L^− 1^ TP in the respective test wells (12 wells total per leachate type per species). Control wells (*n* = 3) received 200 µL of microalgae culture and 800 µL of the modified f/2 medium (based on artificial seawater). Initial cell densities in the wells were approximately 8 × 10^4^ cells mL^− 1^ for *S. costatum*, 5 × 10^4^ cells mL^− 1^ for *Chae. lorenzianus*, 4 × 10^3^ cells mL^− 1^ for *Chat. antiqua*, and 2 × 10^3^ cells mL^− 1^ for *K. mikimotoi*. The initial cell densities for each species were standardized based on in vivo chlorophyll *a* fluorescence to ensure consistent detection by the plate fluorescence reader. The density for *S. costatum* was set to provide a robust initial signal, and other species were adjusted accordingly, accounting for differences in cell size and chlorophyll content. All species remained in the exponential growth phase throughout the test period, without reaching the stationary phase.

Microalgae growth was monitored by measuring in vivo chlorophyll *a* fluorescence in each well using a fluorescence image analyzer (FLA-7000, FUJIFILM, Tokyo, Japan). Measurements were taken four times: 24-hours intervals for the diatoms (*S. costatum*, *Chae. lorenzianus*) and 48-hours intervals for the flagellates (*Chat. antiqua*, *K. mikimotoi*). A pre-established linear relationship between in vivo chlorophyll *a* fluorescence and cell density was used to convert fluorescence readings to cell densities.

The average cell division rates (µ_2_, expressed as divisions day^− 1^) were calculated according to Fukazawa et al. (1980). For diatoms, data from the 0–96 h (0–4 days) interval were used; for flagellates, data from the 96–192 h (4–8 days) interval were used. Growth rate inhibition (I_µ2_) was calculated using the following formula:

I_µ2_ = (µ_2c_– µ_2x_) / µ_2c_.

where µ_2c_ is the mean cell division rate in the control wells (*n* = 3), and µ_2x_ is the mean rate in the wells exposed to a specific leachate concentration (*n* = 3).

### Effects of tire leachate on photosynthetic efficiency

In a separate experiment, the impact of the tire leachates on the photosynthetic activity of the microalgae was assessed using a pulse amplitude modulation (PAM) fluorometer (Water-PAM, Heinz Walz, Effeltrich, Germany). While the basic experimental design (leachate concentrations, controls, replicates) was consistent with the procedures described in the previous section, modifications were made to accommodate the sensitivity of PAM fluorometry. Specifically, the total volume per well was doubled (to 2 mL), and the initial cell density was tripled compared to the growth experiment.

Measurements were performed 24 h after the addition of the test solutions (leachates or control medium). Prior to each measurement, cells were dark-adapted for 15 min. Rapid light curves (RLCs) were generated by exposing the samples to eight incremental steps of actinic light (up to 1800 µmol photons m^− 2^ s^− 1^) for 30 s per step (Higo et al. 2017).

The effective quantum yield of photosystem II (ΦII) at a light intensity comparable to the culture conditions (120 µmol photons m^− 2^ s^− 1^) was extracted from the RLC data for each well. This parameter was used as an indicator of photosynthetic performance under leachate exposure.

The inhibition of photosynthetic efficiency (I_ΦII_) was calculated as follows:

I_ΦII_ = (ΦII_c_ – ΦII_x_) / ΦII_c_.

where ΦII_c_ is the mean yield in the control wells (*n* = 3), and ΦII_x_ is the mean yield in the wells exposed to a specific leachate concentration (*n* = 3).

### Non-Targeted chemical analysis of tire leachate

To determine the major components in each tire leachate, a non-targeted analysis was performed to detect a wide range of organic compounds. Sample preparation was designed to minimize the loss of components. Specifically, the simple pretreatment method consisted of placing 600 µL each of the tire leachate and 40% methanol (as an anti-adsorption agent) in a 2-mL brown glass vial, and mixing by inversion.

Components were analyzed by liquid chromatography-high resolution tandem mass spectrometry (LC-HRMS/MS) using a Vanquish Flex UHPLC system (Thermo Fisher Scientific) coupled with an LTQ Orbitrap XL high-resolution MS/MS system (Thermo Fisher Scientific; Eliuk and Makarov 2015). Chromatographic separation was performed on a COSMOSIL 2.5C_18_-MS-Ⅱ column (2.0 mm i.d. × 100 mm, 2.5 μm, Nacalai Tesque, Kyoto, Japan). The mobile phase consisted of (A) ultrapure water and (B) acetonitrile. The gradient elution program at a flow rate of 0.4 mL min^− 1^ was as follows: starting at 20% B; linearly increased to 90% B from 0 to 35 min; held at 90% B until 40 min; returned to 20% B at 41 min; and maintained at this condition until 50 min for re-equilibration. The column oven and sample cooler were maintained at 35 °C and 4 °C, respectively. The injection volume was 5 µL. Mass spectrometry was performed using positive electrospray ionization (ESI^+^), and high-resolution MS (HRMS) spectra of precursor ions were acquired over an *m/z* range of 80–600. MS/MS spectra of product ions were simultaneously obtained by collision-induced dissociation (CID) using 35% collision energy from major precursor ions at each retention time. Due to the presence of salts derived from the culture medium, the eluent corresponding to retention times between 0 and 5 min was diverted and excluded from MS analysis. The acquired mass spectra were analyzed using Xcalibur Qual Browser software (Thermo Fisher Scientific) to identify the detected compounds.

### Quantification of 1,3-diphenylguanidine (DPG)

Since DPG was suspected to be a major toxic component (see Results), it was quantified following a protocol developed by the Environmental Science Center, Kanagawa Prefectural Government (2023) with modifications to the analytical column, addition of a guard column, and adjustments to the monitoring ions. Detailed protocols for the measurements are given in the supplementary information (Method S1 and Table S1). The recovery rate and method quantification limit (MQL) for DPG were determined as follows: the recovery test was conducted by spiking pretreated environmental seawater samples with DPG at a concentration of 5 µg L^− 1^, followed by analysis using LC-MS/MS. A calibration curve was constructed by analyzing DPG standards at concentrations of 4, 10, 40, 100, 400, and 1000 µg L^− 1^ in methanol, using 10 µg L^− 1^ atrazine-^13^C_3_ as an internal standard. For each analysis, the correlation coefficient (*r*^*2*^) of the calibration curve exceeded 0.999. The recovery test was performed with seven replicates, and the standard deviation (SD) of the measured concentrations and the mean recovery rate were calculated. The average recovery of DPG was 105 ± 4.2%. The MQL was calculated as 10 times the SD of the analyte concentrations obtained in the recovery tests, resulting in a value of 4 µg L^− 1^. The same quantification method was applied to the test solutions described in the following section.

### Marine microalgae exposure experiments with 1,3-diphenylguanidine (DPG)

To evaluate the effect of DPG, a commercially available pure form of 1,3-diphenylguanidine (DPG; CAS No. 102-06-7, Tokyo Chemical Industry, Tokyo, Japan) was used. A primary stock solution of DPG (200 mg L^− 1^) was prepared in artificial seawater (Marine Art SF-1). From this stock solution, a series of working standard solutions (2.5, 5, 10, 20 and 40 mg L^− 1^) was prepared by serial dilution with artificial seawater. These solutions were stored at -20 °C until use. For the exposure experiments, 900 µL of exponentially growing microalgae cultures were dispensed into 18 wells of a multi-well plate. For each concentration level, 100 µL of each working standard solution was added to triplicate wells, resulting in final DPG concentrations of 0.25, 0.5, 1, 2, and 4 mg L⁻¹. Control wells (*n* = 3) received 900 µL of microalgae culture and 100 µL of artificial seawater. To monitor the stability and actual exposure concentrations of DPG during the experiment, a parallel setup was prepared. Because the volumes in the culture wells were insufficient for chemical analysis, corresponding test solutions were prepared separately by mixing the culture medium with DPG. These solutions were maintained under the same incubation conditions as the experimental cultures. Aliquots were collected at the beginning and end of the experiment for DPG quantification. The geometric mean concentrations of DPG were used to estimate the 50% effective concentration (EC_50_). Experimental conditions, including incubation temperature, light regime, and growth inhibition assessment, were consistent with those used in the tire leachate exposure experiments.

### Statistical analysis

Statistical comparisons between the treatment concentrations and the control group were performed using Dunnett’s test, implemented with the multcomp package in R (version 4.4.2; R Core Team 2024). This analysis was based on the growth rate (µ_2_) and effective quantum yield (ΦII). A statistically significant inhibitory effect compared to the control was defined as *p* < 0.001. The No Observed Effect Concentration (NOEC) was defined as the highest tested concentration that did not show statistically significant inhibition (*p* ≥ 0.001). The 50% effective concentrations for growth rate inhibition (EC_50−µ2_) and photosynthetic yield inhibition (EC_50−ΦII_) were calculated using the respective inhibition rates (I_µ2_ and I_ΦII_) derived from each test. For these calculations, inhibition rates ≤ 0 were set to 0.001, and rates ≥ 1 (i.e., ≥ 100%) were set to 0.999. EC_50_ values were estimated based on measured concentrations of the test substance using dose-response modeling with the drc package (Ritz 2010) in R (version 4.4.2). Although the EC_50_ calculations generally followed the procedure outlined in ISO 10253 (ISO 2024), sharp decreases in µ_2_ or ΦII were frequently observed, even between consecutive concentrations in a twofold dilution series. This pattern suggested a potential threshold effect rather than a typical concentration-dependent response. Therefore, both the EC_50_, representing concentration-dependent toxicity, and the NOEC, representing the no-effect threshold, were determined using the results of Dunnett’s test. Measured concentrations during the tests were approximately 89–100% of the nominal values (see Table S2 in the supplementary information), so toxicity values were calculated based on the nominal concentrations. For calculating the toxicity contribution rate of DPG, EC_50−µ2_ values were also determined based on actual concentrations. Only datasets that satisfied the mandatory condition specified in ISO 10253 – “The concentrations should be chosen to obtain at least one inhibition below and one inhibition above the intended EC(r)x parameter” – were used for EC_50_ estimation. However, the additional condition in the same guideline – “Additionally, at least two levels of inhibition between 10% and 90% should be included to provide data for regression analysis” – was often not met. Data points that did not fulfill this condition are marked with an asterisk (*).

To estimate the contribution of DPG to the overall toxicity of the tire leachates, the toxicity contribution rate was calculated. This was done by dividing the predicted toxicity of DPG within the leachate by the EC_50−µ2_ of pure DPG (based on the actual concentration). The predicted toxicity was calculated by multiplying the measured concentration of DPG in the leachate (mg L^− 1^) by the EC_50−µ2_ of the leachate (g L^− 1^), and then dividing this product by the nominal concentration of the leachate (10 g L^− 1^). For this calculation, EC_50−µ2_ values for the tire leachates were used as calculated in R, regardless of whether they met the ISO 10253 condition for reporting, to enable assessment based on the available dose-response data.

### Hemolysis assay with 1,3-diphenylguanidine (DPG)

The hemolytic activity of DPG was evaluated following the method described by Henkelman et al. (2009). DPG stock solutions were prepared in phosphate-buffered saline (PBS) at concentrations of 0.0125, 0.025, 0.05, 0.1, 0.2, 0.4, 0.8, 1.6, 3.2, 6.4, 12.8, 25.6, 51.2, and 104.2 mg L^− 1^. A 10% (v/v) solution of Triton X-100 (CAS 9036-19-5, Wako, Tokyo, Japan) in PBS was used as a positive control, and PBS alone served as the negative control.

Washed red blood cells (RBCs) were prepared from sheep defibrinated blood (027-00013-01, Japan Bioserum, Tokyo, Japan). The blood was mixed with PBS and centrifuged for 10 min at 800 rpm, after which the supernatant was discarded. This washing step was repeated three times.

The assay was conducted in a round-bottom 96-well plate (1-1601-03, AS ONE, Tokyo, Japan). In each well, 270 µL of the washed RBC suspension and 30 µL of either a DPG stock solution, the positive control, or the negative control were added in triplicate. Final DPG concentrations in the wells were 1.25, 2.5, 5, 10, 20, 40, 80, 160, 320, 640, 1280, 2560, 5120, and 10420 µg L^− 1^. The plate was incubated at 37 ± 1 °C for 24 h, and hemolysis was assessed by visual inspection.

## Results

### Inhibition of growth and photosynthesis in four marine microalgae species by crude tire leachates

**Fig. 1:**
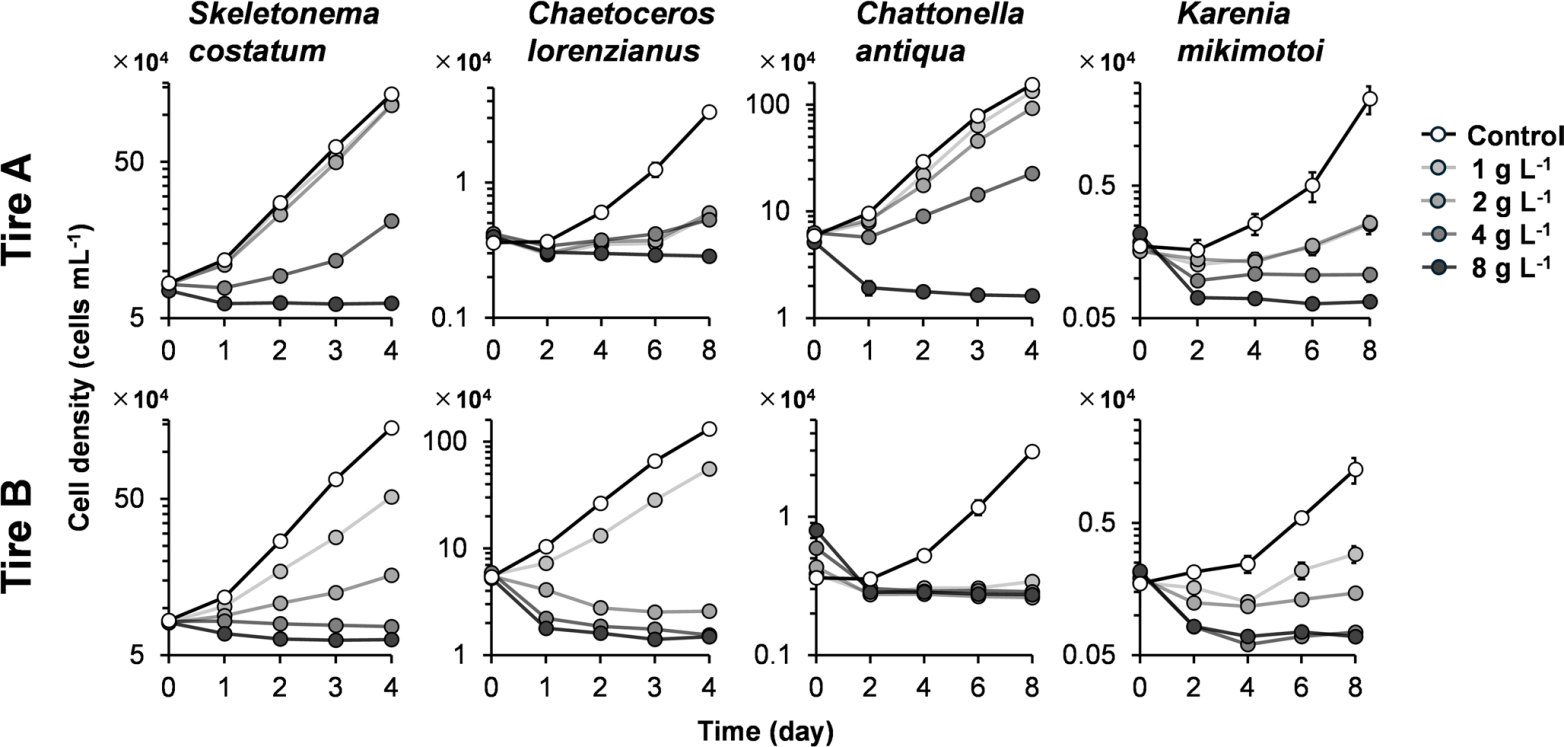
Growth curves of four marine microalgae species: diatoms *Skeletonema costatum* and *Chaetoceros lorenzianus*, a raphidophyte *Chattonella antiqua*, and a dinoflagellate *Karenia mikimotoi*, exposed to leachates from Tire A and Tire B. Final leachate concentrations were equivalent to 1, 2, 4, and 8 g L⁻¹ of tire particles (TP). Control groups received no leachate. Data points represent the means of three replicates, and error bars indicate standard deviation

Figure 1 shows the growth curves of two diatom species (*Skeletonema costatum* and *Chaetoceros lorenzianus*) and two flagellate species (*Chattonella antiqua* and *Karenia mikimotoi*) exposed to various concentrations of the leachates from Tire A and Tire B. Compared to the control groups without leachate addition, both Tire A and Tire B leachates inhibited growth. Among the diatoms (left two panels in Fig. 1), a concentration-dependent decrease in growth was observed. In contrast, the flagellates (right two panels in Fig. 1) exhibited pronounced growth inhibition even at the lowest concentration tested (1 g L^− 1^).

**Fig. 2:**
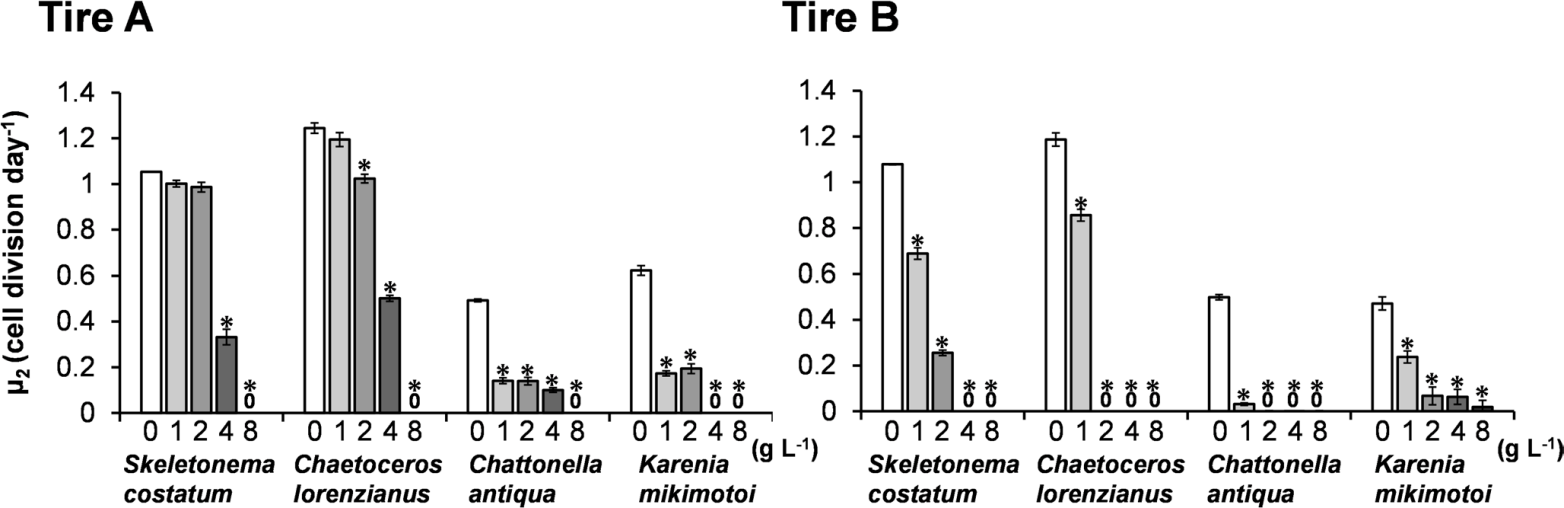
Daily cell division rates (µ_2_) of four marine microalgae exposed to leachates from Tire A and Tire B. Bars represent the mean cell division rates calculated from the 0–4-day interval for diatoms (*S. costatum*, *Chae. lorenzianus*) and the 4–8-day interval for flagellates (*Chat. antiqua*, *K. mikimotoi*). Error bars indicate the standard deviation from three replicates. Asterisks (*) denote statistically significant differences from the control group (*p* < 0.001, Dunnett’s test)

From these growth curves, the daily cell division rate (µ_2​_) for each species was calculated (Fig. 2). For *S. costatum*, the cell division rate was significantly reduced at concentrations of 4 g L^− 1^ and above for the Tire A leachate (*p* < 0.001), and at 1 g L^− 1^ and above for the Tire B leachate. Similarly, for *Chae. lorenzianus*, significant inhibition was observed at 2 g L^− 1^ and above for the Tire A leachate, and at 1 g L^− 1^ and above for the Tire B leachate. The cell division rates of the flagellates *Chat. antiqua* and *K. mikimotoi* were drastically reduced even at the lowest tested concentration (1 g L^− 1^) of both Tire A and Tire B leachates. Overall, Tire B consistently caused lower cell division rates than Tire A at the same concentrations in three of the four species, with the exception of *K. mikimotoi*.

**Fig. 3:**
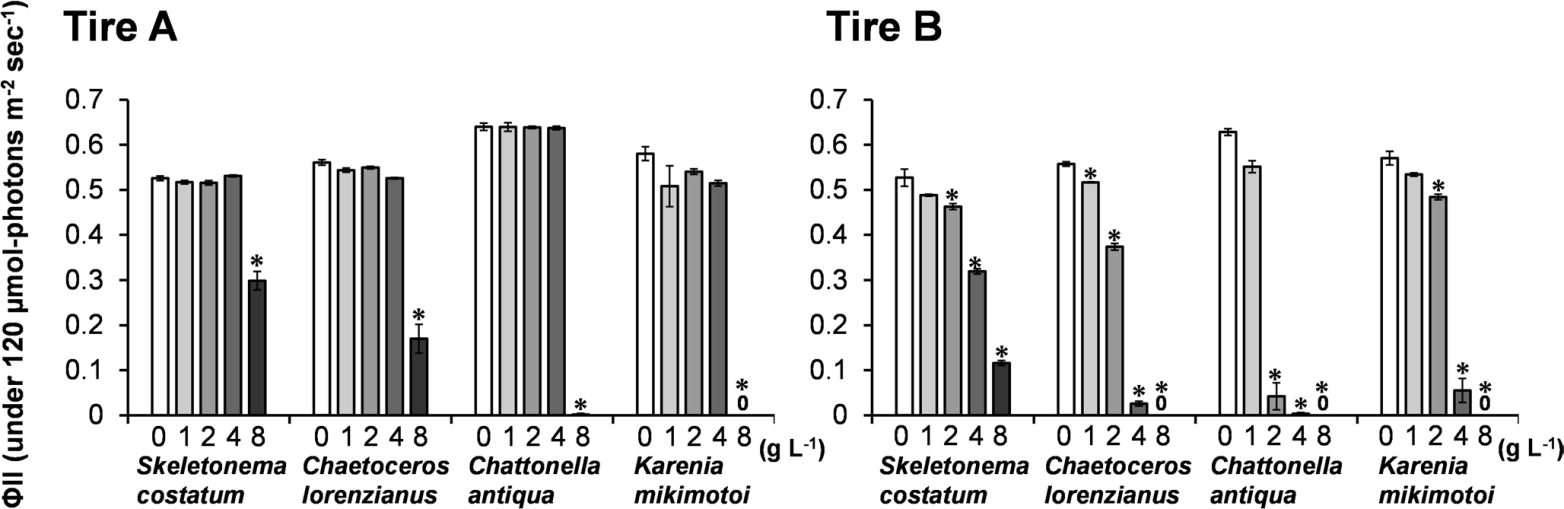
Effective quantum yield of photosystem II (ΦII) in four marine microalgae species after 24-hour exposure to the leachates from Tire A and Tire B. Measurements were taken at a light intensity of 120 µmol photons m^− 2^ s^− 1^. Bars represent the means of three replicates, and error bars indicate standard deviation. Asterisks (*) denote statistically significant differences from the control group (*p* < 0.001, Dunnett’s test)

Figure 3 presents the effective quantum yield of photosystem II (ΦII​), measured at 120 µmol-photons m^− 2^ sec^− 1^, after 24-hour exposure to the leachates from Tire A and B. With the Tire A leachate, ΦII​ significantly decreased (*p* < 0.001) in all four species at the highest concentration (8 g L^− 1^). At this concentration, the relative ΦII​ values (% of control) were: *Chat. antiqua* (0%) < *K. mikimotoi* (0.3%) < *Chae. lorenzianus* (30.3%) < *S. costatum* (56.9%). For the Tire B leachate, ΦII​ significantly decreased in all four species at concentrations of 2 g L^− 1^ and above. At 2 g L^− 1^, the relative ΦII​ values (% of control) were: *Chat. antiqua* (6.7%) < *Chae. lorenzianus* (67.0%) < *K. mikimotoi* (84.9%) < *S. costatum* (87.8%).

EC_50_ and NOEC values for the leachates from Tire A and Tire B were determined using the data on daily cell division rate (µ_2_; Fig. 2) and effective quantum yield of photosynthesis (ΦII; Fig. 3), respectively (Table 1). For the diatoms *S. costatum* and *Chae. lorenzianus*, the Tire B leachate exhibited higher toxicity than Tire A, as indicated by lower EC_50−µ2_ and EC_50−ΦII_ values. In addition, NOEC_−µ2_ and NOEC_−ΦII_ values for Tire B could not be precisely established in many cases, as effects were observed even at the lowest tested concentration (< 1 g L^− 1^), further supporting its higher toxicity. For the two flagellate species, severe growth inhibition caused by both leachates precluded the calculation of EC_50−µ2_ and NOEC_−µ2_. However, based on photosynthetic yield, Tire B again showed higher toxicity, with lower EC_50−ΦII_ and NOEC_−ΦII_ values compared to Tire A.

**Table 1:** The 50% effective concentrations (EC_50_) and no observed effect concentrations (NOEC) for growth rate (µ_2_) and photosynthetic yield (ΦII) inhibition in four marine phytoplankton species exposed to leachates from Tire A and Tire B

	EC_50−µ2_ (g L^− 1^)		NOEC_−µ2_ (g L^− 1^)		EC_50−ΦII_ (g L^− 1^)		NOEC_−ΦII_ (g L^− 1^)	
	Tire A	Tire B	Tire A	Tire B	Tire A	Tire B	Tire A	Tire B
*Skeletonema costatum*	3.50*	1.28	2.0	< 1.0	> 8.0	4.72	4.0	1.0
*Chaetoceros lorenzianus*	3.37	1.09*	1.0	< 1.0	7.50*	2.29*	4.0	< 1.0
*Chattonella antiqua*	< 1.0	< 1.0	< 1.0	< 1.0	4.10*	1.35*	4.0	1.0
*Karenia mikimotoi*	< 1.0	0.97	< 1.0	< 1.0	4.77	2.70*	4.0	1.0

EC50_–µ2_ and NOEC_–µ2_ were calculated from the 0–4-day interval for diatoms (*Skeletonema costatum, Chaetoceros lorenzianus*) and the 4–8-day interval for flagellates (*Chattonella antiqua, Karenia mikimotoi*) Asterisks (*) indicate those that do not meet the following condition specified in ISO 10253: “Additionally, at least two levels of inhibition between 10% and 90% should be included to provide data for regression analysis”

### Chemical analysis of tire leachates

**Fig. 4:**
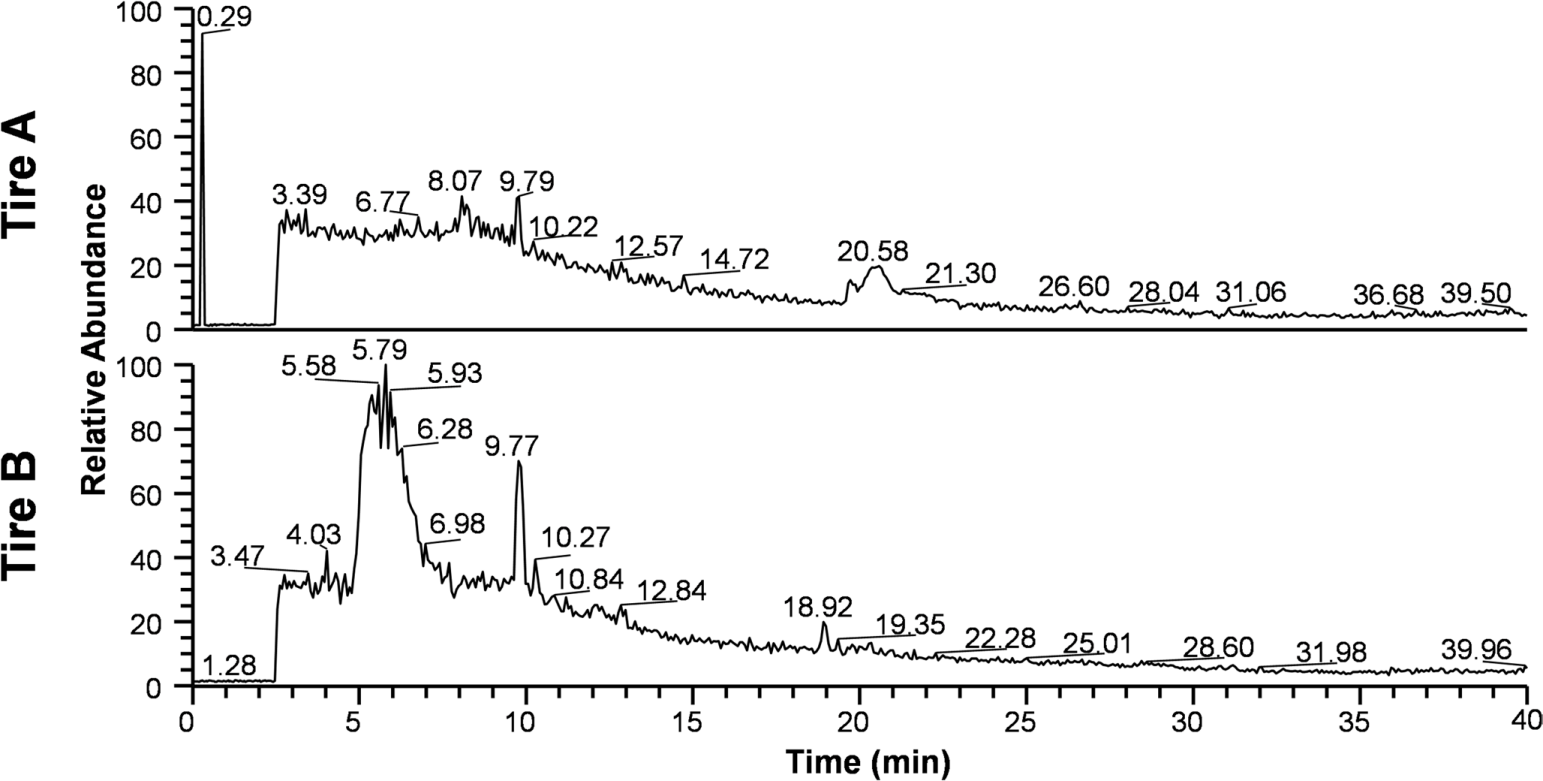
Total ion chromatograms (TICs) of water-soluble leachates from Tire A and Tire B obtained by LC-HRMS analysis. Relative peak intensities are shown with the largest peak set to 100. The prominent peak at a retention time of 5.79 min in the Tire B leachate was identified as 1,3-diphenylguanidine (DPG)

Organic compounds in the leachates from Tire A and B were analyzed using LC-HRMS/MS. Total ion chromatograms (TICs) obtained from LC-HRMS analysis are shown in Fig. 4. The TIC for Tire A leachate displayed a peak at 9.79 min, while the TIC for Tire B leachate showed a prominent peak at 5.79 min and another peak at 9.77 min. According to the high-resolution mass spectrum, the major peak at 5.79 min corresponded to a compound with *m/z* = 212.11810, which closely matched the theoretical *m/z* value of 212.11822 for the protonated molecule [M + H]⁺ of 1,3-diphenylguanidine (DPG) (Δppm = -0.57, within ± 5 ppm). The major fragment ions (*m/z* = 94, 119, 195) observed in the MS/MS spectra were also consistent with previously reported values for DPG (Lin et al. 2007). Based on these findings, the compound was identified as DPG. Quantitative analysis by LC-MS/MS revealed that DPG was not detected in the Tire A leachate (< 0.004 mg L^− 1^), but was present at a concentration of 11.37 mg L^− 1^ in the Tire B leachate.

### Effects of DPG on growth and photosynthesis of four marine microalgae species

**Fig. 5:**
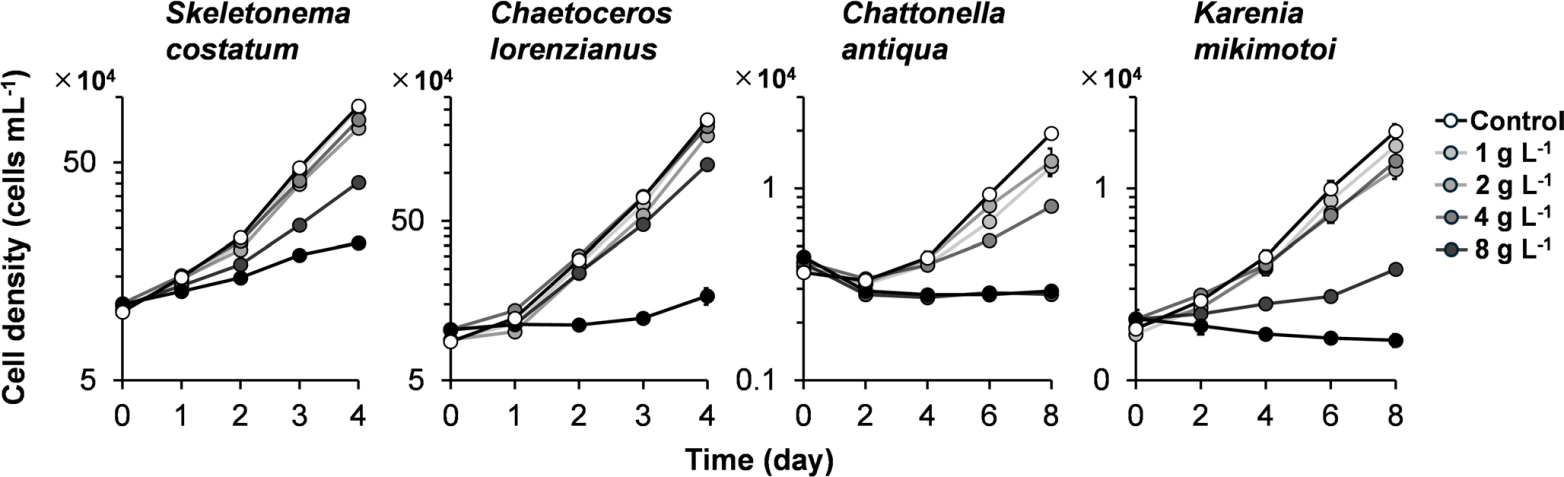
Growth curves of four marine microalgae species exposed to various concentrations of 1,3-diphenylguanidine (DPG). Cells were exposed to DPG concentrations of 0.25, 0.5, 1, 2, and 4 mg L^− 1^. Data points represent the means of three replicates, and error bars indicate standard deviation

Figure 5 shows the growth curves of two diatom species (*S. costatum* and *Chae. lorenzianus*) and two flagellate species (*Chat. antiqua* and *K. mikimotoi*) exposed to various concentrations of DPG. Compared to the control groups (no DPG addition), DPG exposure tended to reduce growth in all four species. At the highest concentration tested (4 mg L^− 1^), growth was markedly inhibited in all species.

**Fig. 6:**
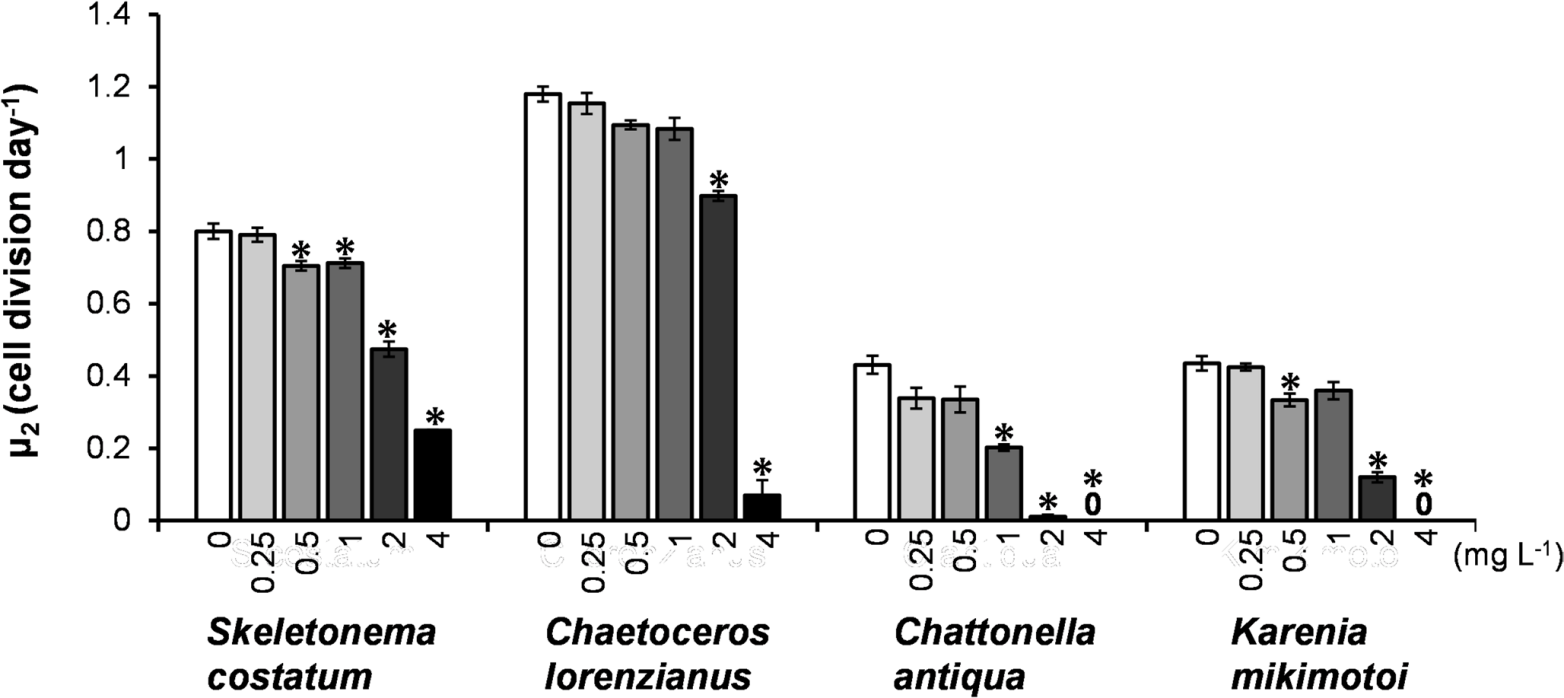
Daily cell division rates (µ_2_) of four marine microalgae exposed to 1,3-diphenylguanidine (DPG). Bars represent the mean cell division rates calculated over the relevant exposure period for each species. Error bars indicate standard deviation from three replicates. Asterisks (*) denote statistically significant differences from the control group (*p* < 0.001, Dunnett’s test)

Based on these growth curves, daily cell division rates (µ_2_) were calculated for each species (Fig. 6). At DPG concentrations of 2 mg L^− 1^ and above, all four species showed significantly reduced cell division rates. The relative cell division rates at 2 mg L^− 1^ DPG (% of control) were as follows: *Chat. antiqua* (2.5%) < *K. mikimotoi* (27.6%) < *S. costatum* (59.2%) < *Chae. lorenzianus* (76.1%).

**Fig. 7:**
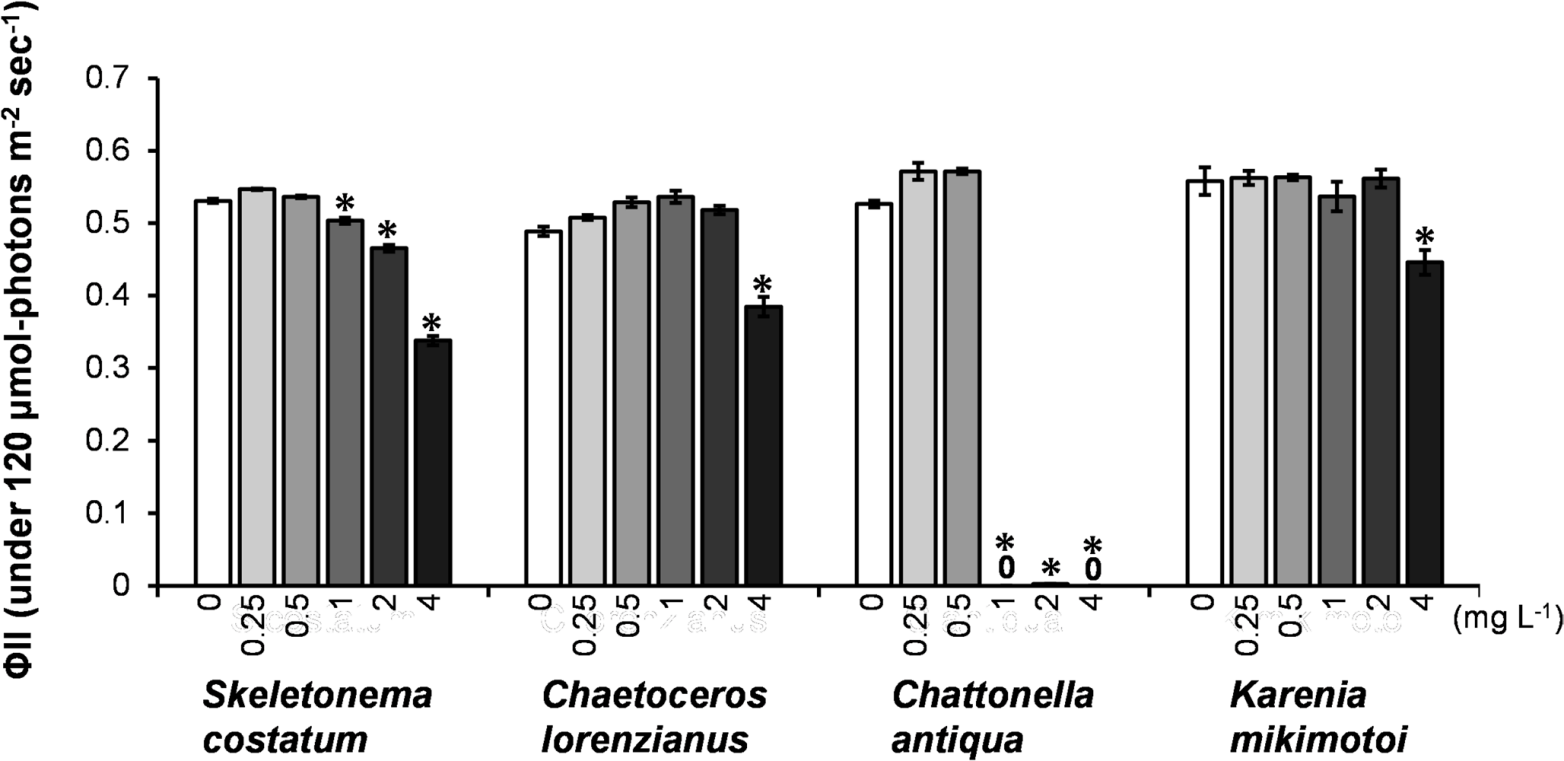
Effective quantum yield of photosystem II (ΦII) in four marine microalgae after 24-hour exposure to 1,3-diphenylguanidine (DPG). Measurements were taken at a light intensity of 120 µmol photons m^− 2^ s^− 1^. Bars represent the means of three replicates, and error bars indicate standard deviation. Asterisks (*) denote statistically significant differences from the control group (*p* < 0.001, Dunnett’s test)

Figure 7 presents the effective quantum yield of photosystem II (ΦII) after 24-hour exposure to DPG. At 4 mg L^− 1^, ΦII was significantly reduced in all species. The relative ΦII values at this concentration (% of control) were: *Chat. antiqua* (0%) < *S. costatum* (63.7%) < *Chae. lorenzianus* (78.7%) < *K. mikimotoi* (79.9%). The ΦII of *C. antiqua* remained similar to the control at 0.5 mg L^− 1^ but dropped sharply at 1 mg L^− 1^, indicating a threshold-like response.

Based on the results for daily cell division rate (µ_2_; Fig. 6) and effective quantum yield (ΦII; Fig. 7), the EC_50_ and NOEC values of DPG for growth (EC_50−µ2_, NOEC_−µ2_) and photosynthesis (EC_50−ΦII_, NOEC_−ΦII_) were calculated (Table 2). The EC_50−µ2_ for *Chat. antiqua* was 0.93 mg L^− 1^, the lowest among the four tested species. Additionally, its EC_50−ΦII_ was estimated as 0.68 mg L^− 1^, while the value could not be determined for the other three species because photosynthetic inhibition did not exceed 50% even at the highest tested DPG concentration (4 mg L^− 1^). These results indicate that *Chat. antiqua* was the most sensitive to DPG among the species examined.

**Table 2:** The 50% effective concentrations (EC_50_) and no observed effect concentrations (NOEC) for growth rate (µ_2_) and photosynthetic yield (ΦII) Inhibition in four marine phytoplankton species exposed to 1,3-diphenylguanidine (DPG)

Skeletonema costatum	EC_50−µ2_ (mg L^− 1^)	NOEC_−µ2_ (mg L^− 1^)	EC_50−φⅡ_ (mg L^− 1^)	NOEC_−φⅡ_ (mg L^− 1^)
	2.54 (2.45)	0.25	> 4.0	0.5
*Chaetoceros lorenzianus*	2.59 (2.50)*	1.0	> 4.0	2.0
*Chattonella antiqua*	0.93 (0.89)	0.5	0.68*	0.5
*Karenia mikimotoi*	1.47 (1.41)	1.0	> 4.0	2.0

The EC_50−µ2_ values in parentheses are based on measured concentration. Asterisks (*) indicate values that do not meet the following condition specified in ISO 10253: “Additionally, at least two levels of inhibition between 10% and 90% should be included to provide data for regression analysis”

The diatoms *S. costatum* and *Chae. lorenzianus* exhibited similar EC_50−µ2_ values of 2.54 mg L^− 1^ and 2.59 mg L^− 1^, respectively. However, their NOEC_−µ2_ values differed significantly, at 0.25 mg L^− 1^ and 1.0 mg L^− 1^, respectively, suggesting that *S. costatum* was affected by DPG at lower concentrations. The EC_50−µ2_ value for *K. mikimotoi* was 1.47 mg L^− 1^, indicating an intermediate sensitivity between *Chat. antiqua* and the diatoms. Overall, based on EC_50−µ2_ values, the flagellates (*Chat. antiqua* and *K. mikimotoi*) appeared more sensitive to DPG than the diatoms (*S. costatum* and *Chae. lorenzianus*).

### Effect of DPG on washed RBCs

**Fig. 8:**
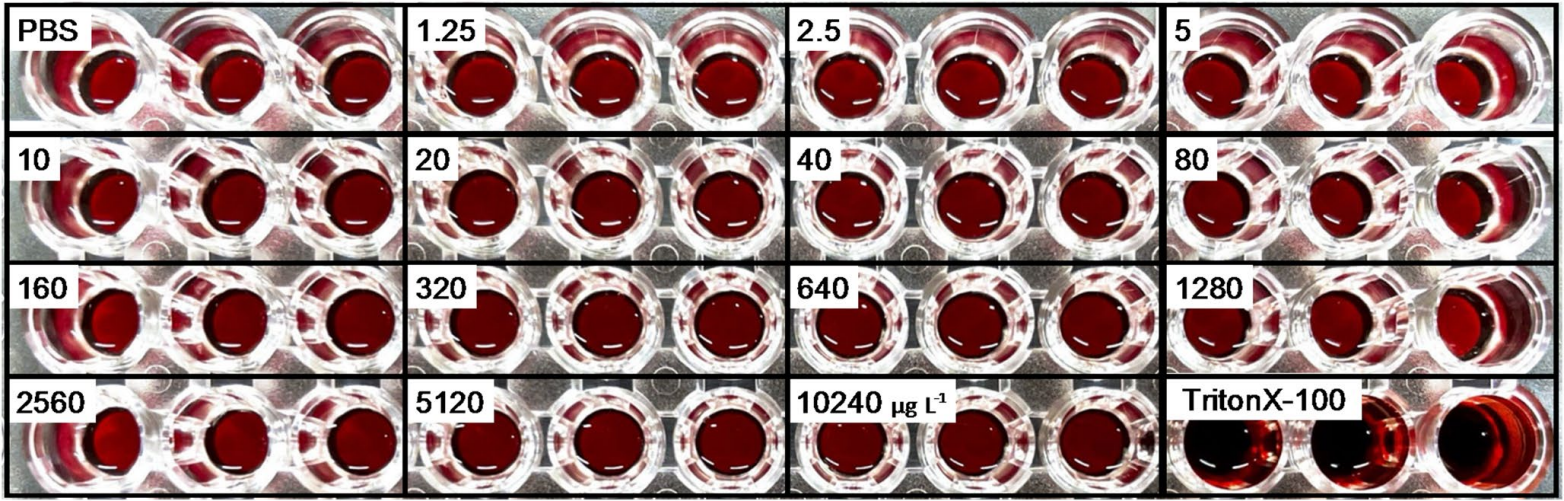
Representative image from the hemolysis assay evaluating the membrane-disrupting potential of 1,3-diphenylguanidine (DPG). Triton X-100 was used as a positive control, and phosphate-buffered saline (PBS) served as the negative control. Final DPG concentrations in the assay wells (triplicate) were 1.25, 2.5, 5, 10, 20, 40, 80, 160, 320, 640, 1280, 2560, 5120, and 10420 µg L^− 1^.

Figure 8 shows washed red blood cells (RBCs) 24 h after exposure to DPG. As illustrated, hemolysis was observed only in the positive control group treated with Triton X-100. DPG did not induce hemolysis at any of the tested concentrations.

## Discussion

### Inhibitory effects of tire leachate on microalgae

In this study, leachates prepared from two different types of tires (Tire A and Tire B) exhibited adverse effects on the growth and photosynthetic activity of all four tested marine microalgae species. The 96-hour EC_50_ values for growth inhibition (EC_50−µ2_) ranged from < 1.0 to 3.50 g L^− 1^. Notably, the raphidophyte *Chattonella antiqua* was the most sensitive species. A general trend emerged in which flagellated algae (*Chat. antiqua* and the dinoflagellate *Karenia mikimotoi*) displayed greater sensitivity to tire leachates than diatoms (*Skeletonema costatum* and *Chaetoceros lorenzianus*) (Tables 1 and 2).

These toxicity values are comparable to those reported for crustaceans. For instance, the 96-h LC_50_ for the copepod *Tigriopus japonicus* exposed to a leachate prepared from 10 g L^− 1^ of tire particles was 5.34 g L^− 1^ (Yang et al. 2022). For the amphipod *Hyalella azteca*, a 48-h NOEC of 0.26 g L^− 1^ was reported (Halle et al. 2021). Furthermore, Bournaka et al. (2023) found LC_50_ values for three copepod species to be in the range of 0.22–3.43 g L^− 1^. The similarity between our EC_50_ values for microalgae and the reported LC_50_ values for crustaceans suggests that both primary producers and consumers at higher trophic levels may be highly sensitive to tire-derived chemicals.

Our findings are also broadly consistent with other studies on marine microalgae. Page et al. (2022) reported 72-h EC_50_ values of 0.23 g L^− 1^ for the dinoflagellate *Heterocapsa steinii*, 0.64 g L^− 1^ for the cryptophyte *Rhodomonas salina*, and 0.73 g L^− 1^ for the diatom *Thalassiosira weissflogii*, with the dinoflagellate being the most sensitive. However, direct comparisons between studies are challenging because the chemical composition and resulting toxicity of leachates can vary significantly depending on the tire type. This variability was evident in our study, where the EC_50−µ2_ values for diatoms differed by approximately threefold between the Tire A and Tire B leachates (Table 1). Chemical analysis confirmed distinct profiles for each leachate (Fig. 4), and as will be discussed, the compound DPG appears to be a key factor driving the toxicity of Tire B.

### Diphenylguanidine (DPG) as a primary toxicant

The 96-h EC_50−µ2_ values for pure DPG against the four microalgae species ranged from 0.89 to 2.50 mg L^− 1^ (Table 2, based on actual concentrations). To evaluate the contribution of DPG to the overall toxicity of Tire B leachate, we compared the EC_50_ values of the leachate and pure DPG, along with the measured concentration of DPG in the leachate. DPG accounted for a substantial portion of the leachate’s toxicity for all species: 59.3% for *S. costatum*, 49.4% for *Chae. lorenzianus*, 75.2% for *Chat. antiqua*, and 78.5% for *K. mikimotoi*. These high contribution rates strongly suggest the identification of DPG as a primary toxicant in the Tire B leachate.

The toxicity of DPG has been documented by the European Chemicals Agency (ECHA), which reports a 96-h EC_50_ of 1.4 mg L^− 1^ for a freshwater microalga *Pseudokirchneriella subcapitata* and a 72-h EC_50_ of 7.5 mg L^− 1^ for another freshwater microalga *Desmodesmus subspicatus*. The 48-h LC_50_ for a freshwater cladoceran *Daphnia magna* was 17 mg L^− 1^, and a recent study reported a 48-h LC_50_ of 13.7 mg L⁻¹ for another freshwater cladoceran *Moina macrocopa* (Yoganandham et al. 2025). The EC_50−µ2_ values obtained in this study (0.93–2.59 mg L^− 1^) are comparable to or even lower than those reported for freshwater species, indicating that marine microalgae are highly sensitive to DPG. Notably, *Chat. antiqua* exhibited extreme sensitivity, with a 24-h EC_50_ for photosynthetic efficiency (EC_50−ΦII_) of just 0.68 mg L^− 1^ (Fig. 7; Table 2).

### Proposed mechanism of action for tire leachate and DPG

As noted, flagellates were more sensitive to the leachates than diatoms. Furthermore, they exhibited different response patterns. Diatoms showed a classic dose-dependent inhibition of growth and photosynthesis, whereas the flagellates, particularly *Chat. antiqua*, displayed a threshold-like response, with toxicity increasing sharply above a certain concentration (Figs. 2, 3, 6 and 7). This aligns with observations by Page et al. (2022), who also found a dinoflagellate to be more sensitive than a diatom. The mechanism of toxicity may involve membrane disruption, although this remains uncertain. We confirmed that DPG did not cause hemolysis of red blood cells (Fig. 8). Instead, we hypothesize that this differential sensitivity is linked to cell surface structure. DPG possesses a guanidine group, which is readily protonated in aqueous solutions to form a positively charged guanidinium cation (OECD 2002). We propose a mechanism similar to that of other guanidinium-containing compounds, such as the disinfectant polyhexamethylene guanidine phosphate (PHMG-P), which exerts cytotoxicity by electrostatically interacting with and disrupting negatively charged cell membranes (Song et al. 2019). It is plausible that the cationic DPG binds to the negatively charged surfaces of microalgae cells, destabilizing the membrane, increasing its permeability, and ultimately leading to cell lysis observed in *Chat. antiqua* at high concentrations (Figs. 6 and 7). The high sensitivity of *Chat. antiqua* may be amplified by its production of an extracellular mucilage layer composed of polysaccharides (Yokote and Honjo 1985; Okamoto et al. 2000). This negatively charged layer could adsorb and concentrate the cationic DPG at the cell surface. In contrast, the rigid silica frustules of diatoms may offer a degree of physical protection against such surface-acting toxicants. This membrane destabilization could also facilitate the intracellular transport of DPG and other organic compounds, potentially leading to oxidative stress, as reported by Jiang et al. (2023) for another alga.

### Environmental presence and potential impact of DPG

DPG is increasingly recognized as an environmental contaminant of concern. It is classified as a Persistent and Mobile Organic Compound (PMOC), meaning it resists degradation and is highly water-soluble, making it difficult to remove from aquatic systems (Ichihara et al. 2023). DPG has been detected widely in German rivers at concentrations in the tens of ng L^− 1^ range, with tire wear identified as the primary source (Zahn et al. 2019). It is consistently found in tire leachate and has been detected at high concentrations (0.52 µg L^− 1^) alongside 6PPD-quinone (2.85 µg L^− 1^) in stormwater runoff during rain events (Johannessen et al. 2022). Its presence in water samples linked to salmon mortality events has further heightened scientific interest (Peter et al. 2018). While tire wear is a major source, DPG may also leach from other polymer materials, such as O-rings and seals used in drinking water systems (Johannessen et al. 2021; Santos and Snyder 2023).

In this study, the Tire B leachate contained a notably high concentration of DPG (11.37 mg L^− 1^). The toxic concentrations identified for marine microalgae (EC_50_: 0.93–22.59 mg L^− 1^, or 930–2590 µg L^− 1^) are several orders of magnitude higher than the environmental concentrations recently reported in Japan’s Arakawa River (1–467 ng L^− 1^) (Xie et al. 2021). Consequently, one recent risk assessment classified DPG as “low risk” based on a comparison of measured environmental concentrations (MECs) and predicted no-effect concentrations (PNECs) (Xu et al. 2024). While reports of DPG in seawater are less frequent than in freshwater, concentrations of 0.28–93 ng L ^− 1^ have been recorded in US and Chinese coastal waters (Tian et al. 2019; Jin et al. 2024; Peter et al. 2024; Zhang et al. 2024). Similar to other tire-derived chemicals, these marine DPG levels are lower than those typically observed in freshwater (Müller et al. 2025); consequently, DPG is classified as posing an even lower risk in the marine environment compared to freshwater. Nevertheless, given its persistence, mobility, multiple sources beyond tires, and the potential to form unknown transformation byproducts during water chlorination, the environmental fate and ecological effects of DPG warrant continued investigation.

### Limitations and future directions

It should be noted that the concentrations of tire particles and DPG used here are higher than average levels in natural seawater. However, this study intentionally focused on establishing a toxicological baseline and estimating the maximum potential hazard to marine microalgae. This approach ensured a clear dose-response relationship for determining EC_50_ values and provided a reference for the maximum hazard potential. Such data are essential for evaluating risks in high-loading areas, such as coastal zones, where localized concentrations may be significantly elevated.

While this study successfully identified DPG as the primary toxicant in the Tire B leachate, the causative agents for the toxicity of the Tire A leachate, which contained negligible DPG, remain unknown. Recent research has implicated cyclic amines as key toxicants in tire leachate for a freshwater microalga (Jiang et al. 2023), suggesting that other compounds from this class may be responsible for the toxicity of Tire A. A further limitation is that this study did not conduct a comprehensive, non-target analysis of all chemicals present. Tire leachates are known to contain numerous other compounds, including 6PPD and its highly toxic transformation product, 6PPD-quinone (Xu et al. 2024), which were not evaluated here. Therefore, future research should focus on two key areas. First, identifying the unknown toxicants in tire leachates like Tire A is crucial for a complete understanding of their environmental risk. Second, the effects of other major tire-derived contaminants, such as 6PPD and 6PPD-quinone, on marine microalgae must be assessed.

Despite these unknowns, the high-production-volume chemical DPG remains a substance of interest due to its persistence, mobility, and widespread use as a vulcanization accelerator in numerous rubber products beyond tires. Additionally, while this study established a toxicological baseline under optimal growth temperatures, future research should incorporate environmental variables such as seasonal temperature fluctuations. Since temperature can influence both chemical leaching rates and algal sensitivity, addressing this factor will provide a more comprehensive and realistic environmental risk assessment of tire-wear particles.

## Conclusion

This study demonstrates that water-soluble leachates from tires inhibit key marine microalgae. We identified the vulcanization accelerator 1,3-diphenylguanidine (DPG) as a primary causative toxicant, contributing 49.4–78.5% of the toxicity in one tire’s leachate. The flagellates tested were more sensitive to DPG and tire leachate than the diatoms, a difference that may be explained by the diatoms’ protective silica frustules, which likely shield the cell membrane from DPG. Toxicity was also found in the leachate without DPG, highlighting the need for future research to identify other toxic compounds, such as cyclic amines and other known tire-related contaminants (e.g., 6PPD).

## Supplementary Information

Below is the link to the electronic supplementary material.

Supplementary Material 1

## Data Availability

Data is provided within the manuscript or supplementary information files.
