# Supplementary material for: Growth Inhibition of marine microalgae by water-soluble extracts from automobile tires and identifying key chemical contributors to toxicity
Source: Ecotoxicology. 2026 Feb 18;35(3):62. doi: 10.1007/s10646-026-03051-6 (PMC12917054; doi:10.1007/s10646-026-03051-6)
Supplement: Supplementary file 1 — Supplementary Material 1 [file 10646_2026_3051_MOESM1_ESM.pdf]

Supplementary Information

Article title

Growth inhibition of marine microalgae by water-soluble extracts from automobile tires and identifying key chemical contributors to the toxicity

Journal

Ecotoxicology

Authors and affiliations

Taisei Fujimura[1,¶], Soichiro Hirashima[1,¶], Toshimitsu Onduka[2,¶], Shizuka Ohara[2,*], Kazuhiko Takeda1, Kazuhiko Koike1

[1]Graduate School of Integrated Science for Life, Hiroshima University, Higashihiroshima, Hiroshima, Japan

[2]Hatsukaichi Branch, Fisheries Technology Institute, Fisheries Research and Education Agency, Hatsukaichi, Hiroshima, Japan

[¶] These authors contributed equally to this work.

[*]**Corresponding author**

Shizuka Ohara

Hatsukaichi Branch, Fisheries Technology Institute, Fisheries Research and Education Agency, Hatsukaichi, Hiroshima, Japan

E-mail: ohara_shizuka02@fra.go.jp

Contents

Supplementary Method: S1

Supplementary Tables: S1, S2

Supplementary Method S1

Detailed protocol for determination of 1,3-diphenylguanidine (DPG) concentrations.

DPG concentrations in the leachates and test solution of DPG exposure experiments were analyzed following methods specified by the Environmental Science Center, Kanagawa Prefectural Government, Japan (2023) with modifications regarding changes in analytical columns, addition of guard columns, and adjustment of monitor ions (Table S1). The test solution for analysis was diluted with ultrapure water as necessary to bring the nominal concentration within the calibration curve range, mixed 1:1 with 1% ammonia methanol, centrifuged at 5,000 g for 2 minutes, and the supernatant was filtered through Millex LG (Merck 0.20 µm, 13 mm) to make the analysis sample. Calibration curve for the chemical was constructed by analyzing chemical standards at concentrations of 4, 10, 40, 100, 400, and 1000 µg L^{-1} in methanol with 10 µg L^{-1} atrazine-$^{13}C_3$ (100 mg L^{-1} nonane solution, 99%, Cambridge Isotope Laboratories, Inc., Tewksbury, MA, USA) as the internal standard using LC-MS. The correlation coefficient for the calibration curves was consistently greater than 0.999 for the analysis. The recovery rate and method quantification limit (MQL) for each chemical were determined as follows: artificial seawater samples (Marine Art SF-1, Tomita Pharmaceutical, Tokushima, Japan) were spiked with chemicals (5 µg L^{-1}) in seven replicates. The SDs of analyte concentrations were calculated, and the mean recovery rates for the pesticides were determined using Equation (1):

$$R = \frac{Cs - Cu}{K} \times 100 \quad (1)$$

where R is the percentage recovery rate (%), Cs is the chemical concentration in the spiked sample (µg L^{-1}), Cu is the pesticide concentration in the non-spiked sample (µg L^{-1}), and K is the known concentration of the spike (µg L^{-1}). The MQL was calculated as 10 times the SD of analyte concentrations in the recovery tests, 4 µg L^{-1} was obtained. The average recovery rate was 105 %.

Reference

Environmental Science Center, Kanagawa Prefectural Government, Japan (2023) 1,3-Diphenyl guanidine (in Japanese). In: General Environmental Policy Bureau, Ministry of the Environment, Japan (ed) Reiwa 3 nenndo Kagakubussitu bunnsekihoukaihatsutyousa houkokusyo (Report on Research and Development of Chemical Analysis in FY2021). Ministry of the Environment, Tokyo, pp 154–172

Supplementary Tables

Table S1. Liquid chromatography–tandem mass spectrometry (LC–MS/MS) conditions for 1,3-diphenylguanidine (DPG) analysis

<table>
<tr><td rowspan="14">LC</td><td>Model</td><td colspan="6">SHIMADZU Nexera X2</td></tr>
<tr><td>Analytical column</td><td colspan="6">Wako Ultra C18-2 column (2.1 mm id × 75 mm, 2 µm)</td></tr>
<tr><td>Guard column</td><td colspan="6">InertSustain C18 guard cartridge (2.1 mm id × 10 mm, 2 µm)</td></tr>
<tr><td>Mobile phase A</td><td colspan="6">1 mM ammonium formate in ultrapure water (pH 9.0)</td></tr>
<tr><td>Mobile phase B</td><td colspan="6">Methanol</td></tr>
<tr><td rowspan="6">Gradient</td><td colspan="6">Water analysis for toxicity test</td></tr>
<tr><td colspan="6">0 min (A:B = 80:20)</td></tr>
<tr><td colspan="6">0–4 min (A:B = 5:95)</td></tr>
<tr><td colspan="6">4–6 min (A:B = 5:95)</td></tr>
<tr><td colspan="6">7 min (A:B = 80:20)</td></tr>
<tr><td colspan="6">7–14 min (A:B = 80:20)</td></tr>
<tr><td>Flow rate</td><td colspan="6">0.2 mL min^{-1}</td></tr>
<tr><td>Column over temp.
Sample cooler temp.</td><td colspan="6">40 °C
4 °C</td></tr>
<tr><td>Injection volume</td><td colspan="6">5 µL</td></tr>
<tr><td rowspan="4">MS/MS</td><td>Model</td><td colspan="6">8030 (Shimadzu)</td></tr>
<tr><td rowspan="3">Monitor ion (m/z)</td><td>Name</td><td>RT (min)</td><td>Ionization</td><td>Quantification</td><td colspan="2">Confirmation</td></tr>
<tr><td>DPG</td><td>6.5</td><td>ESI (+)</td><td>212.00 > 119.05</td><td>212.00 > 94.05</td><td>212.00 > 77.05</td></tr>
<tr><td>Atrazine $^{13}C_3$</td><td>6.7</td><td>ESI (+)</td><td>219.05 > 177.05</td><td>219.05 > 98.05</td><td>219.05 > 70.00</td></tr>
</table>

Table S2. Nominal and measured concentrations of 1,3-diphenylguanidine (DPG) in the exposure tests and their concentration ratios.

Nominal concentration (mg L^{-1})	Measured concentration (mg L^{-1})				Measured concentration/ nominal concentration (%)
	0 d	4 d	8 d	Geometric mean [a]	
0	0.009	<0.004		0.004	-
0.25	0.251	0.232		0.241	97
0.5	0.504	0.458		0.480	96
1	0.913	0.931		0.922	92
2	2.052	1.771		1.906	95
4	3.972	3.905		3.938	98
0	0.009		<0.004	0.004	-
0.25	0.251		0.197	0.222	89
0.5	0.504		0.482	0.493	99
1	0.913		0.94	0.926	93
2	2.052		1.968	2.010	100
4	3.972		3.897	3.934	98

[a] The measured concentration was calculated using half the method quantification limit (0.002 mg/L) when the test chemical was not detected.
